# QuickStats

**Published:** 2015-01-16

**Authors:** FROM THE NATIONAL CENTER FOR HEALTH STATISTICS

**Figure:**
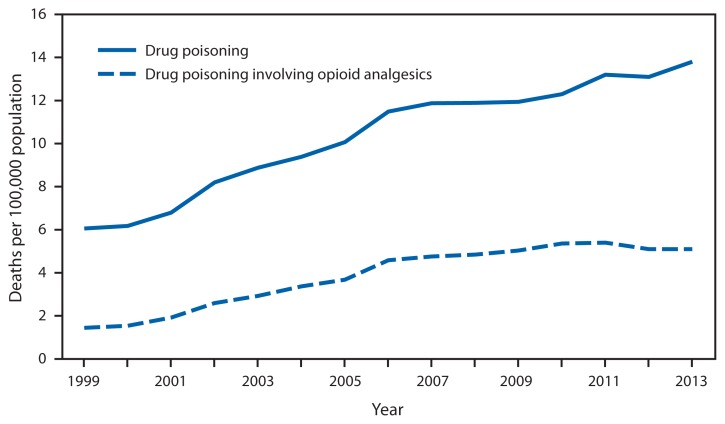
Rates* of Deaths from Drug Poisoning^†^ and Drug Poisoning Involving Opioid Analgesics^§^ — United States, 1999–2013 * Per 100,000 population, age-adjusted to the 2000 U.S. standard population. ^†^ Drug poisoning deaths can result from taking an overdose of a drug, being given the wrong drug, taking a drug in error, or taking a drug inadvertently. Drug poisoning deaths include all intents (i.e., unintentional, suicide, homicide, and undetermined intent). ^§^ Drug poisoning deaths are identified using the I*nternational Classification of Diseases, Tenth Revision (ICD-10)* underlying cause of death codes X40–X44, X60–X64, X85, and Y10–Y14. Drug poisoning deaths involving opioid analgesics are the subset of drug poisoning deaths with a multiple cause of death code of T40.2–T40.4.

In 2013, a total of 43,982 deaths in the United States were attributed to drug poisoning, including 16,235 deaths (37%) involving opioid analgesics. From 1999 to 2013, the drug poisoning death rate more than doubled from 6.1 to 13.8 per 100,000 population, and the rate for drug poisoning deaths involving opioid analgesics nearly quadrupled from 1.4 to 5.1 per 100,000. For both drug poisoning and drug poisoning involving opioid analgesics, the death rate increased at a faster pace from 1999 to 2006 than from 2006 to 2013.

**Sources:** National Vital Statistics System mortality data. Available at http://www.cdc.gov/nchs/deaths.htm.

Chen LH, Hedegaard H, Warner M. Drug-poisoning deaths involving opioid analgesics: United States, 1999–2011. NCHS data brief no. 166. Hyattsville, MD: US Department of Health and Human Services, CDC; 2014. Available at http://www.cdc.gov/nchs/data/databriefs/db166.htm.

**Reported by:** Li-Hui Chen, PhD, eyx5@cdc.gov, 301-458-4446; Holly Hedegaard, MD; Margaret Warner, PhD.

